# News and Notes

**Published:** 1900-08

**Authors:** 


### *NEWS AND NOTES.*

THE *Memphis Lancet* and the *Memphis Monthly* have become consolidated under the title of the last mentioned journal.

DR. BENTON STRANGE of Swainsboro, Ga., died in that city on July 11th. Dr. Strange was one of the most prominent physicians in his section.

A SHAKER.—Doctor: "Did you shake well before using?"

Larry (who has had chills): "Phoy, Docthor, Oi tuk th' medicine to kape me from shakin'."—*Chicago News.*

SHOCK.—"Skinner got a bill the other day for his wife's automobile drives, and he's been laid up ever since." "What's the matter?" "The doctor says he's suffering from an overcharge of electricity."—*Life.*

THE committee charged with the organization of a collective investigation of cancer in Germany is now fully formed. Professors von Leyden and Kirchner are the presidents, and Dr. George Meyer is the secretary.

PROFESSOR A. S. GRUNBAUM, of Vienna, claims priority in the discovery of the agglutinating effect of serum on typhoid bacilli, known as Widal's reaction, his claim being based on a certificate signed by Professor Nothnagel and Dr. Mannoberg.

DR. JNO. ASHHURST, JR., of Philadelphia, died in that city on July 7th. Dr. Ashhurst was the author of a text-book on surgery which has probably proven the most popular work on that subject in the various medical schools that has ever been written.

---

THE coming of hot weather is not welcomed by Londoners (the thermometer has registered over 90° F. this week), and a general exodus is beginning. It is feared that another spell like that of last year is impending. Until the beginning of this week London's death-rate, thanks to the cool weather, was the lowest in seven years—13.5 in 1,000.

---

THERE has been established in Paris a Bureau of American Trained Nurses, the list consisting chiefly of graduates from the Presbyterian and St. Luke's Hospital of New York. It is under the direction of Mrs. W. H. Booth, 102 Rue Vaugirard, Paris, France. This will render it possible to secure good nurses for Americans on the "other side."

---

DR. ALEXANDER J. C. SKENE of Brooklyn, N. Y., died at his summer home on the Cattskill on July 6th. Dr. Skene was one of the most prominent gynecologists of this country, and his text-book on this subject has long been an acknowledged authority. Dr. Skene was conservative, and yet displaying rare judgment when operative skill was necessary.

---

CATCHING.—Mamma: "What is Willie crying about?"

Bridget: "Shure, ma'am, he wanted to go across the street to Tommy Green's."

Mamma: "Well, why didn't you let him go?"

Bridget: "They were having charades, he said, ma'am, and I wasn't shure as he'd had 'em yet."—*Exchange.*

---

AN aeronaut was recently poisoned by hydrogen arsenide which escaped from the balloon. This shows the necessity of purifying the hydrogen arsenide used for balloon purposes. The balloon was filled in the ordinary way, and nothing peculiar in the odor of the gas was noticed. A few hours afterwards persons who had assisted in the operation were taken seriously ill.

---

THE "Solace," which, since the Spanish war, has been used as a transport, has been reconverted, by order of Rear Admiral Remey, into a hospital ship and detailed to carry sick and wounded American soldiers and sailors from China to the hospitals in Japan. The "Relief" has also been ordered from Manila to China. The "Maine" left Plymouth, England, last week for Chinese waters.

---

THE AMERICAN ACADEMY OF MEDICINE.—At the annual meeting of this Society, held at Atlantic City, on June 2d and 4th, the following officers were elected for the ensuing year: President, Dr. Samuel D. Risley, of Philadelphia. Vice-Presidents, Drs. C. M. Culver, of Albany, New York; Rosa Engell, of Chicago, Ill.; G. G. Groff, of San Juan, Porto Rico; Charles T. McClintock, of Detroit, Mich. Secretary and Treasurer, Dr. Charles McIntyre, of Easton, Pa. Assistant Secretary, Dr. Alexander R. Craig, of Columbia, Pa.

---

THE London School of Medicine for Women has just issued its report for 1899. A list is appended of 254 medical women it has trained. They seem now to be called to the farthest ends of the earth to serve in hospitals and as medical missionaries. Nearly every important town in England has a qualified woman doctor. China claims a large number; even Persia claims one, and South Africa has several. Among the different posts held by women doctors are those of medical examiner of a life assurance company, and two female staffs of post-offices. Women are medical inspectors to high schools for girls and children, and boarded out under the Church of England Society for Waifs and Strays; they are lecturers to the London School Board, to the Church Army, and to the Local Government Board.

---

INTERNATIONAL MEDICAL CONFERENCES.—During the Exposition a large number of scientific men will come to Paris and will profit by our International Medical Congress to make known their discoveries and original ideas. But at these meetings the time is very limited, and not more than a few minutes can be allowed each speaker. For this reason we have organized at the International Hospital, 95 Boulevard Arago, a conference service, where both French and foreign physicians can, without charge, hold their lectures, consultations or clinics, taking as much time as is necessary for their demonstrations. A large number of physicians are already registered.

Our confrères are requested to furnish the title of their communication to the organizer of the international conferences, Dr. S. Bernheim, 9 rue Rougemont, Paris, France.

---

THE HILLS ARE GREEN FAR-AWAY.—"Here is the strangest thing in the world," says the *Wichita Eagle*, in a local item from Kingman paper: "A. J. Bittner went to Wichita yesterday to consult a specialist in regard to his eyes." Local item in Wichita paper: "K. L. Brown left yesterday for Chicago to take treatment from an eye specialist there. He fears he may lose his sight." Local from Chicago paper: "R. J. Beaver, president of the Sixth Street National Bank of this city, left yesterday for New York to have Professor J. Agnew, the famous New York specialist on the eye, treat his eyes, which have been troubling him." Local from New York paper: "J. Peter Stuyvesant, of Fifth Avenue, sailed on the Kaiser Wilhelm yesterday for London, where he will be under treatment for the eyes, of S. J. C. Kavenaugh, S.N.A.B." Local from London paper: "The Rt. Hon. Percy Fitzhugh Bighton is sojourning in Paris, under the treatment of the great oculist, Lemaitre Trebon."

